# Dataset of nutrient content and regionalized climate change impacts of food items per consumer country and life cycle stage based on supply location

**DOI:** 10.1016/j.dib.2025.112309

**Published:** 2025-11-22

**Authors:** Christie Walker, Stephan Pfister

**Affiliations:** Institute of Environmental Engineering (IfU), Laura-Hezner-Weg 7, CH-8093 Zurich, Switzerland

**Keywords:** GHG emissions, Regional, Life cycle impact assessment, Distribution, Nutrition, Cooking, Processing, Storage

## Abstract

This dataset quantifies the climate change impacts of approximately 500 food items depending on country of consumption and month consumed, with impacts changing based on the country of cultivation, transportation required, processing necessary, and energy used for storage and home cooking. Country specific crop cultivation impacts were used, where available, from existing databases. These cultivation impacts were combined with product dependent transportation impacts (depending on transport temperature and speed requirements) to the country of consumption. In the case that the original raw product was processed, the energy impacts for the country of origin were used. Various processing methods were incorporated (i.e. freezing, dehydrating, canning). If, after processing and transport to the country of consumption, a food item required refrigeration or freezing during storage before being purchased by the consumer, consumption country specific electricity impacts were included in the food item’s total impact. Once purchased, if the food item would require cooking, the impacts of home cooking were included.

This allowed for a novel dataset that provides impacts and nutritional information for each food item depending on where it is cultivated, how it is processed, and where the final consumer is located, while considering seasonal availability of fresh products each month. It allows for the impacts of individual food items to be directly compared, taking into account their full life cycle. Using this dataset, one can compare the impacts of importing out-of-season fruits and vegetables versus the additional impacts incurred due to processing and long-term storage of the same, locally grown product. As an example – in Switzerland fresh, Swiss grown apricots are only available during limited months, with a certain cultivation impact, and if consumed in Switzerland, a limited transportation impact. Fresh apricots can be imported from other countries, with their own country specific cultivation impacts, outside of these months, with additional transport impacts. Similarly, Swiss produced apricots can be deep frozen (using the Swiss electricity mix) and stored for year-long availability. This dataset offers a way to directly compare the impacts across food items and supply chains, including trade-offs between fresh out-of-season imports and locally produced items, as well as processed alternatives. In addition to the final life cycle impact values, this dataset shows the percentage of contribution of each life cycle stage of a food item to identify hotspots. It offers high reuse potential for researchers, policymakers, and supply chain analysts seeking to assess the temporal and spatial sustainability of food consumption, with the potential to include nutrition as a component. To our understanding, no dataset highlighting the contribution of each life cycle stage for food products, as well as how these contributions change depending on season, trade, and production and consumption countries exist.

Specifications Table

**Table:** 

Subject	Earth & Environmental Sciences
Specific subject area	The dataset shows the country specific climate change impacts for food item consumption including the contribution of all major life cycle stages with a breakdown of the impacts per life cycle stage. This includes the country specific impacts associated with production of the food item, impacts due to transport between the country of production and that of consumption, as well as impacts due to processing, storage, and home cooking, as they are applicable. All impacts are based on country specific emission factors in which the activity would take place. Nutrition content of each food item is included as well.
Type of data	Processed Table (zipped files each in an .xlsx format)
Data collection	Data was collected from a combination of published food impact datasets and literature and compiled using python. Datasets included region specific food inventory and impact values for cultivation, as well as energy use for processing, storage, and home cooking, evaluated using regional electricity emission factors to calculate impacts. A transport model was developed to calculate transport impacts from one country to another, and everything was combined to develop this dataset. Python and Brightway software were used.
Data source location	Data were collected online in Zurich, Switzerland and stored at ETH. Institution: ETH City: Zurich Country: Switzerland
Data accessibility	Repository name: Zenodo Data identification number: 10.5281/zenodo.16318008 Direct URL to data: https://zenodo.org/records/16318008
Related research article	C. Walker, S. Pfister, and S. Hellweg, “Methodology and optimization tool for a personalized low environmental impact and healthful diet specific to country and season,” Journal of Industrial Ecology, vol. 25, no. 5, pp. 1147–1160, 2021. [1] https://onlinelibrary.wiley.com/doi/10.1111/jiec.13131i

## 1 Value of the Data

• The data [2] offers calculated values for the total climate change impacts incurred during each life cycle stage of ∼500 different raw and processed food items depending on country of production and country of consumption for each month of the year. This aggregates several food inventory and impact databases to consider all relevant life cycle stages of food production to consumption, and how impacts differ depending on seasonal availability and the electricity emission intensities of the different countries.
• This dataset is valuable because no existing dataset includes the full life cycle of each food product as it would vary between different countries, while including the same product processed and stored in varying ways (e.g. fresh vs. frozen).
• This dataset serves as a fundamental resource for researchers to link foods to nutrient content (adjusted to include nutrient losses during processing), and impacts, which makes this dataset ideal for optimizing diets to include foods high in nutrients and low in impacts. It is based on a peer-reviewed paper [1] to build personalized and optimized diets.
• Making this data openly available is a relevant benefit for science and industry working on food carbon footprints, identifying life cycle hotspots, and justifying the consumption of local versus imported food items. This also provides a dataset in which one can compare impacts and nutrient content of different food items.

## 2 Background

This dataset was originally developed to support a tool that could be used to optimize diets for both low climate change impacts and high health on a personal level. This tool required both nutrient information and climate change impacts for each food item. Personalization of the diet also required information that would identify an individual’s specific nutrient requirements. Diets were further personalized, however, by using the individual’s country and month of consumption to more accurately calculate the food’s impacts. This led to a dataset with food nutrient content as well as climate change impacts disaggregated by country and month that was used to power the diet optimization tool. As the published paper looked at the optimal diet output for only two countries as case study examples, the full dataset has not been calculated previously. It is therefore unpublished, but as it is a valuable resource to other LCA and diet practitioners, is now available to the public to be used to power other analyses.

## 3 Data Description

There is a total of 85 country-specific excel files provided, with each file representing one consumer country (labeled using the 2-digit ISO code). In each excel file, there are 12 separate sheets, one for each month, and labeled accordingly (i.e. January, February, March, April, May, June, July, August, September, October, November, December.)

Each sheet contains ∼500 identical food items (both raw and processed) listed for the various countries that produce the food item in the specific month. For each food item, the column GHG_Impact_total (kgCO2eq per gram) shows the food item’s total climate change impact – representing the combined greenhouse gas emissions from all life cycle stages involved in producing one gram of that item. This value includes the combined total impact from various life cycle stages, including cultivation/production, transport, processing, home cooking, and storage impacts (freezing and refrigeration calculated separately). The individual contribution of each step, as a percentage of the total impact, is presented in columns B through H. Each identical food item has several possible climate change impacts, for different source countries (indicated in column I – Production Country), because along the life cycle, impacts will vary depending from where the food item was sourced and processed. As an example, in January, cabbage can be produced in 44 countries. Each country has specific production impacts and processing impacts (if applicable) due to different country-specific electricity emission factors. Transportation impacts from the country of production to the country of consumption will vary depending on the distance, temperature, and mode of transport required for distribution. These factors all contribute to varying climate change emissions in the final country of consumption for the same food item, depending on different source countries. Climate change impacts due to storage and home cooking (if applicable) will remain consistent for each food item regardless of production location, as these impacts are dependent on the country-specific electricity emission factors of the country consuming the food (indicated in column J – Consumption Country). An example of how each dataset is populated is shown in Table 1.

**Table 1:** Example dataset setup for apricots consumed in August in Spain, representing the possibility of imported and in-country produced items. Total impact is expressed in kgCO2equivalents per gram of apricot. All other values represent the percentage of the impact that can be attributed to the total impact. Production country indicates the country in which the apricot was processed and/or produced/cultivated.

Food Item	Production	Transport	Processing	Home Cooking	Freezing	Refrigeration	Total Impact	Production Country
Apricots, fresh	30 %	70 %	0 %	0 %	0 %	0 %	0.00035	Italy
Apricots, fresh	100 %	0 %	0 %	0 %	0 %	0 %	0.00015	Spain
Apricots, frozen	9 %	21 %	53 %	0 %	16 %	0 %	0.00112	Italy
Apricots, frozen	23 %	0 %	49 %	0 %	28 %	0 %	0.00066	Spain

There is additionally an excel file (titled ‘Food_Nutrients’) representing the detailed nutrient content of one gram of each food item in the dataset.

## 4 Experimental Design, Materials and Methods

This data was compiled using a combination of data sources and methodologies that are described briefly in the manuscript [1] of the related research article, for which the dataset was originally developed. Additional details are provided here.

Food and nutrient database:

The food items and nutrient content were primarily taken from McCance and Widdowson’s Composition of Foods Database [3] and were supplemented with the USDA Food Database [4] to include detailed fish nutrient content. The McCance and Widdowson’s database was edited to remove composite food items made of multiple ingredients or homemade items, allowing to focus on single ingredient food items that could be combined by the individual to provide a personalized diet. One of the intentions of the tool was to provide information on whether a certain food item would have a lower impact and differing nutrient content depending on whether it was processed for long term storage (such as drying, canning, freezing), or whether it was better to be consumed fresh. As the database contained limited preserved food items, these were manually added to allow for a direct comparison of a food item’s processed impact compared to its raw, unprocessed impact. For example, corn can be consumed as fresh (no processing impacts but limited seasonal availability), canned, or frozen (year-long availability). Canned and frozen corn were each added as a food item to the database (with adjusted nutrient content) to allow for the (country specific) additional impacts of food preservation on a food item.

Country-specific food production and monthly availability of fresh foods:

For each food item in this dataset there are several countries that can potentially produce each item in a particular month. To determine which countries could produce each item, and when, the water demand schedule applied by Pfister and Bayer was used [5]. Their dataset provided a list of crops, countries, and monthly water demand. From this, we determined which countries were able to produce which food items, and in which month these food items would be available. Harvest time of each food item was shifted forward by two months to account for the month for which this item would be available for consumption. Seasonal availability was only applied to fresh products (e.g. fresh apricots); items that were processed for extended shelf life (e.g. canned, frozen, or dehydrated apricots) were assumed to be available year-round.

Seasonality was included to compare the climate change emissions of a particular food product consumed in a country. As an example, the impact of apricots may vary considerably throughout the year depending on where the apricots are sourced. Fresh apricots must be imported from further away during off-season months, thus increasing the impacts associated with the additional transportation. By incorporating this aspect, one can see that in winter months in Switzerland, local fresh apricots will not be available, and as such must be imported from a country currently producing fresh apricots. However, Swiss grown apricots that have been frozen for long term storage will be available, with the added impacts due to freezing and frozen storage of these apricots.

Food item life cycle stage impacts:

There are many stages in the life cycle of a food item that result in climate change impacts, most notably cultivation (i.e. production), transport, processing, cooking, and storage. Food losses were not considered separately in this analysis. The methodology and data sources used to model the impacts at each life cycle stage are detailed below.

### 4.1 Food production

Food production impacts were based on climate change impacts provided in already existing food databases, including a combination of regionalized ecoinvent 3.10 [6] inventories that were evaluated using IPCC 2013 GWP 100 methodology that relates radiative forcing of different greenhouse gases compared to CO_2_ over 100 years (updated from the earlier 3.5 version on which the original paper was based), Clune et al. [7], FAO’s GLEAM [8], Pelletier et al. [9], and Parker et al. [10], and impacts calculated from the Biotrails research project [11]. Impacts for raw food items were taken from the databases directly. Additional impacts were added for processed food items in which processing was not already included, with the methodology described below. Because of the variation in both nutrition and environmental impact, fish were broken down into both wild caught and aquaculture-based species.

Impacts were not always available for each country that could potentially produce each food item. If country specific impacts were available, they were used to represent impacts for that particular food item and country. If a food item was produced in a country in which there were no country specific impacts available, a Rest-of-World or global average was used as a proxy.

### 4.2 Transport

Transport impacts were based on a combination of modes (sea freight, truck, or air transport) and required transit temperatures (no temperature control, refrigerated, or frozen). First, for each food item, all possible countries of production (based on Pfister et al. [5]) and their most likely transport method to the consuming country were evaluated. The most likely transport method was determined based on the proximity of the countries and type of food product being transported. If the production and consumption countries were on the same continent, it was assumed that they were transported only by road, with the distance between country centroids used as a proxy for total travel distance. This distance was calculated using the python package geopy [12]. As this value represents a straight line and doesn’t necessarily indicate true driving distance, it was increased by 20 % to more approximate indicate travel distance [13]. The assumption that land-based transport is preferred to other modes was based on previously published research [14]. If production and consumption countries were on different continents, it was assumed that a portion of their transport was by either cargo ship or air, and a portion was via road. The distance traveled by cargo ship was measured from the port nearest to the production country to the port nearest the consumption country as well as the road distance from the outgoing port to the center point of the production country and from the receiving port to the center point of the consuming country. These distances (both shipping routes and road distances to ports) were based on the CERDI sea database [15]. Flight distances were computed using the great-circle distance function from the python package geopy [12], calculated between the geographic centroids of the producing and consuming countries. It was assumed that specific perishable commodities – such as berries, leafy vegetables, asparagus, and tropical fruits including mangoes and papayas (with bananas excluded) – require rapid transport. Consequently, for intercontinental trade of these items, air freight was assumed in place of sea transport. In-country transportation was not included in the impact calculation, meaning for items produced in the country of consumption, transportation was not considered. We recognize that in the case of larger countries in particular, this assumption may underestimate the product’s total impact. However, based on the provided data, it is expected that this assumption will not heavily influence the final results.

To account for additional impacts regarding temperature regulation during transport, items requiring refrigeration or freezing were modeled according to the impacts associated with refrigerated or frozen means of transport, respectively. It was assumed that all meat and fish were frozen and thawed upon arrival at the destination country. Fresh food and dairy items were assumed to be transported in refrigerated conditions, frozen items in freezing conditions, and all other items such as canned or dried goods in uncooled conditions. Ecoinvent 3.10, using the Brightway software [16], was used to model the various transport impacts.

### 4.3 Processing

Processed items, whose impacts were not already built into the production data from the impact databases that were used, were included as a life cycle stage and added to the total impacts. Processing electricity and steam use was modeled for various processes based on literature values. Each food item requiring industrial processing was categorized into a certain type of processing: 1) bread, 2) pasta, 3) breakfast cereals, 4) large fish canning, 5) small fish canning, 6) general canning, 7) freezing, and 8) dehydrating. For each of these eight processes, the average inputs in the form of electricity and steam were calculated and applied to all corresponding food items.

Processing impacts were modeled based on the electricity mix of each of the producing countries as well as the global impact to produce the necessary steam for the processed food items, according to ecoinvent 3.10 [6]. Items such as milk (both dairy and vegan), cheese, and other meat products had the processing impacts built into the production impact, thus it was not modeled separately.

### 4.4 Home cooking

All food items that would potentially require home cooking before consumption were identified (cooked vegetables and potatoes, rice and other grains, pastas, cooked meat, fish, eggs, and meat substitutes) and these associated impacts were added to the food item’s final impact. Cooking energy required was based on Carlsson-Kanyama et al. [17], and a value indicating the estimated energy required per gram of food item requiring cooking (assumed to be the same globally) was used to estimate impacts. There was no differentiation between personal cooking preferences (e.g. roasting versus boiling) or food item types, with the exception of dried beans. Cooking requirements for dried beans were modeled separately because of their extended cooking time requirements [18]. The energy used for cooking was assumed to be electricity, and the impact associated with this electricity use was modeled based on the electricity impacts in the country of consumption [6], regardless of the production location of the food item. The use of gas or wood as cooking fuel was not modeled.

### 4.5 Storage and retail display

Impacts due to temperature-controlled storage were modeled based on the electricity use from both long-term temperature-controlled storage and the cooling required for the supermarket display. The maximum recommended long-term storage time was modeled for frozen items, with the assumption that items can be stored for an average of 10 months without a loss in quality. Different display requirements were used for different food items, with fresh meat, milk, cheese, frozen fruits and vegetables being modeled separately based on published storage times and electricity use. Impacts from long-term storage of fresh fruits and vegetables were not considered, but a sensitivity analysis of this was considered in the related publication [1].

### 4.6 Final impact

For each food item, impacts from each of the life cycle stages were added for a final kgCO_2_equivalent (GWP100) per gram of food item, with impacts varying depending on production country and country of consumption. The dataset provides these impacts as a final total value, as well as the percentages of the impact that can be attributed to each life cycle stage for each item. As an example, in Spain in August, fresh apricots produced in Italy or Spain (among other countries) may be available for purchase (Table 1). 30 % of the total impact of Italian apricots, consumed in Spain, is due to the Italian specific cultivation/production, and 70 % of the impact is due to transportation from Italy to Spain. In the case of Italian produced frozen apricots, consumed in Spain, production accounts for 9 %, transportation 21 %, processing (assuming an Italian electricity mix) 53 %, and (Spanish) freezing storage 16 %. The contribution of each life cycle stage changes for Spanish produced apricots due to a combination of different production impacts, the reduced transport impacts, and different electricity mix emission factors in the two countries contributing to processing impacts.

## Limitations

Limitations include the limited country specific production impacts provided in the available datasets. When country specific impacts were available, they were included, however when country specific impacts were not available, Rest of World or average impacts were used for each specific producing country. Other limitations include the availability of seasonality in food item production. For crops, seasonality was based on shifted monthly water needs, as these have been used in previous work based on generic crop calendars [5]. However, availability of more accurate datasets representing actual monthly crop harvesting times instead of estimates based on monthly water needs would more accurately capture the monthly, country-specific crop availability. In addition, the inclusion of the effects of greenhouse production, which is largely independent of open-field crop calendars [19], would add another element to this analysis. The impacts of local greenhouse production (and the associated energy use [20]) could be weighed against the transport impacts associated with off-season international trade. Overall, variability and uncertainties in global crop production models are relatively high, as also highlighted in the uncertainty assessments of ecoinvent. Improved differentiation in home cooking modelling, including cooking duration, method, and fuel-type used would offer more accurate impacts than those included here. Regardless of these limitations, this dataset offers insight into the relative importance of life cycle stages of various food items depending on from where they are sourced and where they are consumed.

## Ethics Statement

Our study does not involve human subjects, animal experiments, or any data collected from social media platforms, and we confirm that our research strictly adheres to the guidelines for authors provided by Data in Brief in terms of ethical considerations.

## Data Availability

ZenodoDataset of Nutrients and Regionalized Climate Change Impacts Depending on Country of Consumption for Common Food Items (Original data).
